# Penetrance and pleiotropy in *ATXN2*-related amyotrophic lateral sclerosis

**DOI:** 10.1038/s41431-025-01882-1

**Published:** 2025-06-02

**Authors:** Andrew G. L. Douglas

**Affiliations:** 1 Oxford Centre for Genomic Medicine, Oxford University Hospitals NHS Foundation Trust, Oxford, UK; 2 Nuffield Department of Clinical Neurosciences, University of Oxford, Oxford, UK

**Keywords:** Genetics research, Motor neuron disease

Amyotrophic lateral sclerosis (ALS) is a condition that in recent years has slowly but surely shifted its position along the continuum line between non-genetic and genetic disease. ALS used to rarely be thought of as a genetic disease but since the discovery of *SOD1*-related ALS in 1993, more and more genes linked to the condition have been identified. Furthermore, the proportion of people with ALS found to carry pathogenic variants in these genes (both with and without a family history of ALS) has steadily increased, albeit that the total proportion of affected individuals with such variants remains a minority. However, what do we really mean by calling a disease “genetic” and how much risk of neurodegenerative disease is actually conferred by the growing lists of linked genes and variants? Our previously well-understood ideas of Mendelian inheritance in monogenic disease have become increasingly blurred in this regard owing to issues of highly variable penetrance, expressivity and pleiotropy. In this issue, Demaegd and colleagues add fuel to the fire of this complexity for ALS linked to *ATXN2* repeat expansions [1].

*ATXN2* is an ubiquitously expressed gene present at chromosomal locus 12q24.12. It encodes ataxin-2, a multifunctional protein with roles in RNA metabolism, stress granule formation, endocytosis, calcium signalling, cell metabolism and circadian rhythm [2]. A CAG repeat expansion within the first exon of the gene is known to cause the autosomal dominant condition spinocerebellar ataxia type 2 (SCA2) in individuals carrying 34 or more repeats (full-length expansions). However, smaller repeat sizes of 31-33 (intermediate expansions) have been associated with cases of ALS [3]. Such ALS cases have tended to be sporadic without any apparent family history of the condition, whilst familial cases of ALS have previously only rarely been linked to *ATXN2* [4–6]. In their study, Demaegd and colleagues have identified 10 different familial ALS pedigrees across their cohorts with either intermediate or full-length *ATXN2* expansions [1]. Furthermore, phenotypic pleiotropy is evident in several of these families, leading the authors to suggest the new descriptive term of *ATXN2*-related neurodegeneration, which includes not only SCA2 and ALS but also parkinsonism and essential tremor.

**Fig. 1:**
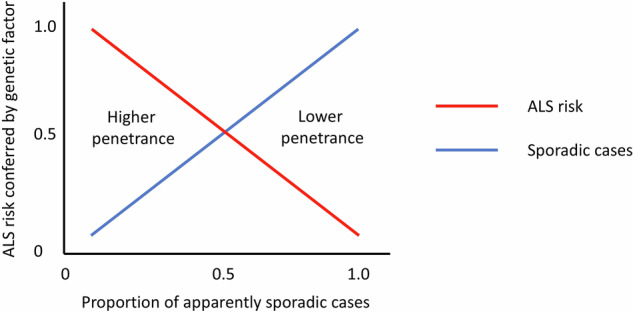
Genetic risk factors and disease penetrance in apparently sporadic ALS. Illustration of the relationship between the absolute disease risk conferred by a genetic risk factor for ALS and the likelihood of observing that risk factor in an apparently sporadic case of ALS. Factors associated with higher disease penetrance will tend to give rise to familial cases and only rarely be found in sporadic ALS. Conversely, factors associated with lower disease penetrance will more commonly be seen in sporadic ALS and will rarely be seen in familial ALS.

The tendency for *ATXN2* expansions to associate predominantly with sporadic but not familial ALS has always been somewhat surprising for a gene otherwise known to cause a monogenic disease (SCA2). The implication of such an observation is that the associated risk of ALS conferred by the expansion genotype cannot be very high when looked at from a population level. If an inherited heterozygous risk factor for a late-onset condition confers high absolute risk then it will tend to cause a dominant phenotype and will tend to be detected more commonly in the setting of familial disease, whilst being comparatively rarer in apparently sporadic disease. Conversely, a genetic risk factor associated with modest absolute risk will only rarely be seen segregating in a dominant fashion with familial disease and is more likely to appear in apparently sporadic cases (see Fig. 1). In this study, 2.5% of a cohort of over 2200 ALS patients tested through a commercial laboratory had 31 or more repeats in *ATXN2* and 0.4% had 34 or more [1]. Only two of these cases were documented as familial ALS, which may suggest that perhaps as few as 3.5% of affected individuals with *ATXN2* expansions have familial ALS.

Another interesting aspect of this study is the assessment of CAA interruptions within the *ATXN2* CAG repeat expansion. Such interruptions have been described in other triplet repeat disorders and are often associated with increased stability of the relevant expansion, which has also been reported as being the case in *ATXN2* [7]. Interestingly, the presence of such interruptions in the cohort of Demaegd et al. did not appear to determine whether or not ALS was the presenting phenotype in this study [1]. If such interruptions are indeed stabilising to the repeat, this finding could suggest that somatic instability of the *ATXN2* repeat may not be the driving factor for disease, at least with regards to ALS.

This research adds to our knowledge about *ATXN2* and the clinical effects of its repeat expansion. Given the evidence presented, the gene will likely need to be thought of clinically not just as a modifying risk factor for disease but also as a potentially disease-causing gene in its own right. However, the overall risk of ALS in people with an *ATXN2* expansion is unlikely to be as high as with some of the more widely prevalent ALS-associated genes like *SOD1* or even the *C9orf72* repeat expansion, which is itself associated with variably reduced disease penetrance [8]. Demaegd and colleagues calculate an odds ratio of 6.5 for having a full-length *ATXN2* expansion in ALS patients compared to controls. This is not quite the same thing as saying there is a 6.5-fold increase in ALS risk but very roughly (and with many caveats) there may be some equivalence between these figures. If so, given the general population lifetime risk of around 1 in 350 for ALS, a 6.5-fold increase in risk above population level would only equate to around 1 in 54 lifetime risk. Such odds would not make testing for the *ATXN2* expansion in isolation a clinically useful test in the setting of an unaffected relative seeking predictive genetic testing for ALS. However, an interesting and important follow-on study will be to see if this level of increased risk conferred by *ATXN2* expansions applies to individuals with known pathogenic mutations in other ALS-linked genes.

While not directly comparable in terms of repeat sizes, a recent population-scale study of repeat expansions in the 100,000 Genomes Project and TOPMed datasets detected carrier frequencies of 1 in 2221 for 33-34 *ATXN2* repeats and 1 in 5136 for 35 or more repeats [9]. These figures are consistent with *ATXN2* expansions having a low ALS disease penetrance at population level when compared to known ALS epidemiology and the proportion of cases found to have *ATXN2* expansions. Importantly, however, the familial cases reported here by Demaegd et al. confirm that ALS disease penetrance can vary substantially between families, even if such cases of highly penetrant disease with *ATXN2* are rare overall. This variability needs to be assessed on an individual family basis when providing genetic counselling to at-risk relatives.

A notable limitation of this study is the relatively small number of families (three) in which segregating genotype data were available. In the remaining families, the cosegregation of the *ATXN2* expansion with disease can therefore only be assumed. Caution must be applied here as families with discordant segregation between disease and risk genotype have been described in ALS, notably with *C9orf72* repeat expansions [10]. Lack of segregation data is unfortunately a common difficulty facing genetic research into late-onset neurodegenerative conditions, where preceding generations with affected relatives will often have already passed away. This can be partially mitigated by ensuring alternative genetic causes are not present. However, whilst this was possible to some extent here, a wide range of different genetic testing strategies were employed across different individuals in this study. Some had extensive analysis of known ALS genes, while others only had testing of the common *C9orf72* repeat expansion and in one family only *ATXN2* itself was analysed. This variability is perhaps inevitable in multicentre observational studies of this kind where testing depends on what is available locally to clinicians and researchers. It is, however, also an argument for considering greater international standardisation with regards to what constitutes appropriate and comprehensive genetic testing in ALS.

This study also highlights a persistent difficulty facing researchers looking for modifying genetic factors in variably penetrant monogenic disorders. Cohorts and registries of patients with ALS (and other conditions) often generate extensive and detailed genomic and phenotypic data on affected individuals. However, those same registries rarely collect rich family history data that includes details of unaffected relatives, even though many such relatives will also be gene carriers. This makes disease penetrance very hard to study. To add to this, studies of families with dominant pedigrees of ALS or related conditions like frontotemporal dementia may often have extensive family history data but in turn lack rich genomic data, since only targeted testing of the known familial variant is generally undertaken. Only if and when both these types of data collection are combined will we finally be able to start to answer questions about how and why penetrance and pleiotropy vary in affected and unaffected individuals carrying the same disease-causing variants.
